# Novel discovery in roles of structural variations and RWP-RK transcription factors in heat tolerance for pearl millet

**DOI:** 10.1007/s44154-023-00092-3

**Published:** 2023-05-15

**Authors:** Bingru Huang, Haidong Yan, Min Sun, Yarong Jin

**Affiliations:** 1 Department of Plant Biology, Rutgers University, New Brunswick, NJ 08901 USA; 2 Department of Genetics, University of Georgia, Athens, GA USA; 3 College of Grassland Science and Technology, Sichuan Agricultural University, Chengdu, China

**Keywords:** Heat stress, Pearl millet, Structural variations, RWP-RK

## Abstract

Global warming adversely affects crop production worldwide. Massive efforts have been undertaken to study mechanisms regulating heat tolerance in plants. However, the roles of structural variations (SVs) in heat stress tolerance remain unclear. In a recent article, Yan et al. (Nat Genet 1–12, 2023) constructed the first pan-genome of pearl millet (*Pennisetum glaucum*) and identified key SVs linked to genes involved in regulating plant tolerance to heat stress for an important crop with a superior ability to thrive in extremely hot and arid climates. Through multi-omics analyses integrating by pan-genomics, comparative genomics, transcriptomics, population genetics and and molecular biological technologies, they found RWP-RK transcription factors cooperating with endoplasmic reticulum-related genes play key roles in heat tolerance in pearl millet. The results in this paper provided novel insights to advance the understanding of the genetic and genomic basis of heat tolerance and an exceptional resource for molecular breeding to improve heat tolerance in pearl millet and other crops.

## Introduction

Global warming with elevated temperatures poses a major threat to food security worldwide (Zhao et al. 2017). Developing heat-tolerant crops is crucial to ensure agricultural production and meet the increasing food demands of a growing population. A thorough understanding of plant heat tolerance mechanisms is essential to improve stress adaptation and mitigate yield loss due to increasing temperatures.

Approaches to unraveling stress tolerance mechanisms include analyzing the natural variation of a broader range of plants for sequence variations showing rapid changes in response to heat stress (Verslues et al. 2023) and integrating transcriptomics, comparative genomics, population genetics, and molecular biological technology (Yan et al. 2023). Structural variations (SVs) indicate large genomic alternations, which contribute to the plant’s phenotypic changes and shape the responses to environments. Recent studies have been carried out to reveal the roles of SVs in the responsiveness of stressful conditions in plants. Fuentes et al. (2019) found the stress response genes were enriched on the rice genomic regions with frequent SVs. Bayer et al. (2019) and Dolatabadian et al. (2020) revealed that disease-resistance genes show diverse SV patterns among different *Brassica* accessions which seems to be a common feature of plant pangenomes. Pan-genomes are becoming necessary to identify the SVs through constructing multiple reference-quality genome assemblies via identifying loci with variants represented by alternative sequences. Recent studies have constructed pan-genomes in major crops, such as maize (*Zea mays*), rice (*Oryza sativa*), tomato (*Solanum lycopersicum*), and soybean (*Glycine max*) (Wang et al. 2018, 2023; Liu et al. 2020), and provided a number of candidate SVs and their surrounding genes linking to important agricultural traits, but the roles of SVs in heat tolerance are un-characterized.

This article highlights the novel findings reported in a recent publication by Yan et al. (2023) which constructed the graph-based pan-genome in pearl millet and revealed the critical roles of SVs contributing to heat tolerance in pearl millet. They used a graph-based pan-genome analysis of pearl millet to identify genetic variations associated with heat stress adaptation and domestication. The information from this article is very useful for genetic improvement and molecular breeding to develop heat-tolerant germplasms of other crops, particularly grass species.

Pearl millet is the sixth most important cereal crop after rice, wheat (*Triticum aestivum*), maize, barley (*Hordeum vulgare*) and sorghum (*Sorghum bicolor*). It is grown in 30 million ha area in the semi-arid and arid tropical regions of Asia and Africa and is a staple food for more than 90 million poor people. Pearl millet is considered as an ideal model crop to study abiotic stress due to its unique characteristics, such as the following: 1) It is a C4 plant species with high photosynthetic efficiency; 2) It can thrive and maintain high and productivity in a wide range of adverse environments, particularly hot and dry conditions. This species can endure high temperature up to 42℃ during reproductive stage (Satyavathi et al. 2021). Recent study have revealed potential molecular mechanisms underlying abiotic stresses (Sun et al. 2020; Khan et al. 2023); however, gene resources of pearl millet are still lacking, and majority of excellent genes or regulatory elements responsive to abiotic stress are unexploited.

**Fig. 1:**
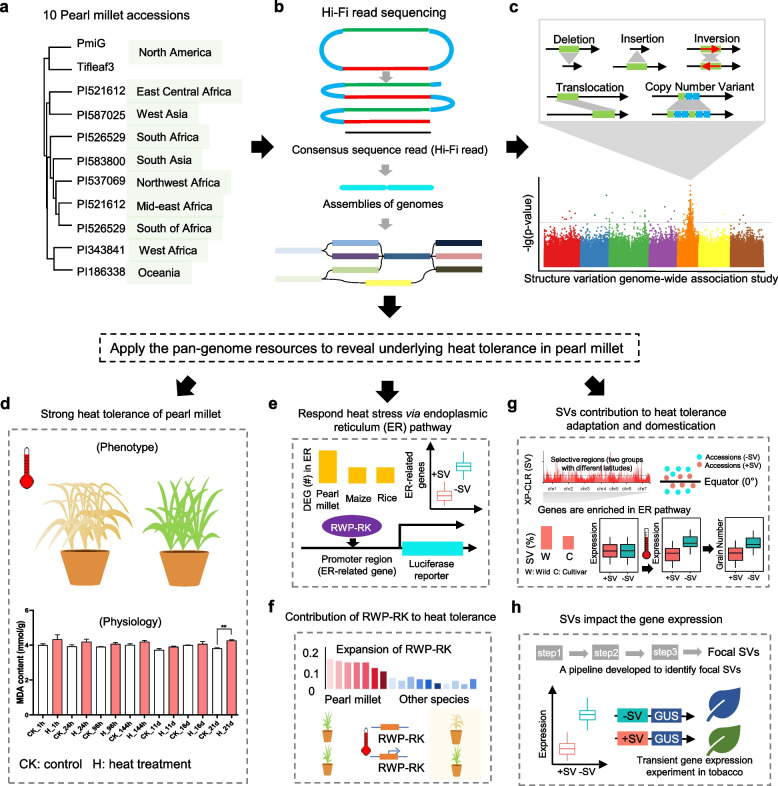
A schematic model exhibiting main results in Yan et al (2023) study. **a** Ten pearl millet accessions are derived from geographically representative regions. **b** PacBio HiFi sequencing was applied to build high quality assemblies that were further used to build a graph-based pan-genome. **c** Five types of SVs were identified in the pan-genome and they could be used as markers linking important agricultural traits. **d** Evaluate heat tolerance of pearl millet based on phenotype and physiology indicators. **e** Transcriptome analysis revealed differential expressed genes (DEGs) involved in endoplasmic reticulum (ER) pathways. **f** RWP-RK TF family was identified to be expanded compared to other species and a transgenic analysis was applied to reveal contribution of RWP-RK to heat tolerance. **g** SVs were found to contribute to heat tolerance adapation and domestication through selection sweep analysis between two accessions groups located in tropical and temperature zones and between cultivar and wild accessions. **h** A pipeline was developed to filter focal SVs potentially affecting nearby gene expression which was further confirmed through a transient gene expression experiment in tobacco

Yan et al. (2023) successfully constructed a pan-genome of pearl millet, including 10 new high-quality genomes and one previously released genome (Fig. 1a, b), and identified 744,364 SVs, most of which were presence and absence variations (PAVs; 622,584), following by 91,852 copy number variations (CNVs), 27,751 translocations (TRANSs), and 2,177 inversions (INVs) (Fig. 1c). The SVs were further categorized into private SVs that were present in only one accession, and the PAVs accounted for 74.70% of private SVs but constituted relatively high proportion (87.51%) of the non-private SVs. Similar trends were detected in CNVs and TRANSs. The quality of pan-genome relies on assembly of each genome as the low quality-assemblies with incorrect orders of contigs of repeats would introduce false positive SVs. Pearl millet has a complex genome with more than 70% sequences were repetitive sequences (Varshney et al. 2017), which increases difficulties to assemble genomes. Yan et al. (2023) utilized a recent technology called PacBio HiFi sequencing with accuracy of 99.9% similar to accuracy via short read and sanger sequencing and have a long read comparable to traditional long read sequencing (Fig. 1b). The authors utilized different approaches to evaluate the assemble qualities per genome on basis of different evaluation scores to prove each assembly having a good quality. They also evaluated the representativeness of the pan-genome, and compared the distribution of SNPs between the 11 accessions and the published 394 core lines. The results showed a similar pattern across the genome and showed strong significant correlations in SNP density, nucleotide diversity and synonymous and nonsynonymous substitution rates. These results indicate the constructed pan-genome are representative of the diversities of the pearl millet population, providing high-quality pan-genome resources for downstream analyses.

Pan-genomes were shown to be applied to identify genetic variations, gene expression patterns, and gene functions. For instance, a pan-genome of 26 soybean accessions was constructed to identify the genetic basis of domestication and adaptation (Liu et al. 2020). Another study on maize pan-genome demonstrated that SVs accounted for a significant proportion of the genetic variation, and identified genes related to flowering time, kernel weight, and starch synthesis (Wang et al. 2023). Yan et al. (2023) identified 4,411 frequency-differentiated structural variations (fdSVs) between tropical and temperate accessions associated with 269 selection sweep regions and 1,471 genes. They also identified 3,952 fdSVs and 1,285 genes associated with domestication. The researchers next conducted a genome-wide association study to examine the associations of the SVs and SNPs with grain number and identified 142 SVs associated with 20 traits (Fig. 1c). Moreover, they analyzed the tiller trait (tiller number/plant) and found four significantly associated SVs near six genes that were not identified based on the SNP data, revealing additional hidden genetic variations that may not be represented by SNPs. These results used several examples to show potential utilizations of the pan-genome that could be used to advance our understanding of contributions of SVs to plant stress adaption and domestication.

Yan et al. (2023) further clarified molecular mechanisms underlying the heat tolerance by integrating transcriptomics, comparative genomics, population genetics, and molecular biological technology. They evaluated heat tolerance of pearl millet and performed RNA sequencing (RNA-seq) under heat stress on two organs at eight-time points and selected six accessions to perform the RNA-seq under two-time points (Fig. 1d, e). They found that differentially expressed genes from the two transcriptome data set were mainly enriched in endoplasmic reticulum (ER) related pathways involved in the repair and elimination of misfolded proteins (Zhu 2016). The protein folding process in the ER is a necessary stress response pathway that can repair uncorrected protein folding caused by high temperature stress (Liu et al. 2012). High temperature induced misfolding peptide chains are recognized and processed by three calnexin and calreticulin, and the genes encoding them are upregulated and differentially expressed in the transcriptome data reported in Yan et al. (2023). These results highlight an essential role of ER system in defense of the heat resistance.

Through comparative genomic analyses, Yan et al. (2023) identified an expanding RWP-RK transcription factor family (Fig. 1f), and found that early LTR expansion may have contributed to the expansion of this transcription factor family in pearl millet. To confirm the role of RWP-RK genes in heat tolerance, they overexpressed one of these genes (*PMF0G00024.1*) in rice and found that the resulting transgenic plants were more tolerant to high temperatures, as manifested by higher levels of antioxidant enzymes (peroxidase and superoxide dismutase) and lower levels of damage-indicating molecules (malondialdehyde) in the transgenic plants than wild-type plants. The researchers further revealed potential associations between the RWP-RKs and the ER-related genes and used transient co-expression to prove the *PMF0G00024.1* could transactivate two important ER-related genes. These findings provide a new clue to indicate potential roles of RWP-RK during heat stress responsiveness, and experimentally confirmed they could interact ER-related genes. The RWP-RKs are also reported mainly involved in nitrate starvation responses (Amin et al. 2023), indicating the RWP-RKs might be a bridge to connect heat tolerance and nitrate signal transduction pathways, and further studies need to be done to uncovered this relationship.

Yan et al. (2023) further examined whether the SVs were involved in stress repair in the ER system. Through selection sweep analysis, this study genotyped SVs by mapping all of the re-sequences to the graph-based pan-genome and focused on SVs with population frequency differences (fdSVs) between accessions from tropical and temperate zones by applying a sliding window methodology. In total, 269 selection sweep regions harboring 4,411 fdSVs surrounding 1,471 genes were identified. And 27 of these genes were significantly and functionally annotated as belonging to ER-related pathways (Fig. 1g). In addition, this study developed a pipeline to systematically identify and validated several focal SVs associated with heat-related gene expression (Fig. 1h). These abovementioned results indicate SVs indeed were involved the expression of some genes in the ER-related pathways contributing to heat tolerance. Although SVs have been identified to be responsive to environmental stress and defence response (Fuentes et al. 2019; Bayer et al. 2019 and Dolatabadian et al. 2020), the SVs underlying heat tolerance were not detaily characterized in these studies. Yan et al. (2023) revealed the SVs potentially particicpate in important processes in the ER system, extending our understanding of roles of SVs underlying heat tolerance.

In summary, Yan et al. (2023) developed the first pan-genome and identified key SVs affecting expression of their nearby heat-responsive genes linked to heat tolerance and domestication for pearl millet. Through an integrated approach, they uncovered a novel TF family RWP-RK that plays an essential role in plant tolerance to heat stress, and functionally validated one member in the major crop rice, enabling improved heat tolerance of the transgenic plant. The results of this study emphasized roles of SVs in abiotic stresses and represent a major advancement in understanding SV-environment interactions and shed light on the molecular mechanisms underlying heat tolerance in pearl millet. The novel findings of RWP-RK provides insights and genetic and genomic basis of genetic modification and molecular breeding for heat-tolerant crops that are better able to withstand high temperatures, which are becoming increasingly important as the climate changes.

## Perspective

The availability of the pearl millet genome resource provides a valuable tool for advancing crop improvement efforts in the face of increasing climate change-induced heat stress. This study has provided massive candidate heat stress-related genes and potential nearby SVs affecting these genes, which assist community to clone key genes in pearl millet or contribute to other major crop improvement through genetic engineering. Also, the genome resource is applicable to be a reference for mutant library in pearl millet. By comparing the genomes or re-sequencing data of the mutant plants with desirable traits to the pan-genome, researchers can identify specific mutations particularly for SVs that are responsible for the observed phenotypes. This application is achievable in any pearl millet panel through mapping their whole-genome sequences to the pan-genome to identify SV-phenotype links. In summary, the availablility of the pearl millet genome sequence opens up new avenues for research aimed at improving heat tolerance in this important crop.

## Data Availability

Not applicable.

## Abbreviations

ER: Endoplasmic reticulum
fdSVs: Population frequency differences
RWP-RK: Transcription factors
TF: Transcriotion factor
SNP: Single nucleotide polymorphism
SVs: Structural variations
